# Case report: Eighteen nails gun shots in the head, thorax and abdomen but still conscious at admission: A challenging patient for the cardiac surgeon!

**DOI:** 10.3389/fcvm.2023.1081162

**Published:** 2023-03-10

**Authors:** Ahmed Ouda, Anna Maria Schürner, Martin Oliver Schmiady, Gerrolt Nico Jukema, Paul Robert Vogt, Thierry Carrel

**Affiliations:** ^1^ Department of Cardiac Surgery, University Hospital, Zurich, Switzerland; ^2^ Institute of Anesthesiology, University Hospital, Zurich, Switzerland; ^3^ Department of Trauma Surgery, University Hospital, Zurich, Switzerland

**Keywords:** penetrating cardiac trauma, head injury, cerebral bleeding, anticoagulation, surgical treatment

## Abstract

We report an unusual case of multiple penetrating cerebral, cardiac and abdominal injuries following a suicidal attempt using a nail gun. Successful treatment required several emergency procedures and resulted from a wise interdisciplinary management and timing of surgery.

## Case report

**Figure 1:**
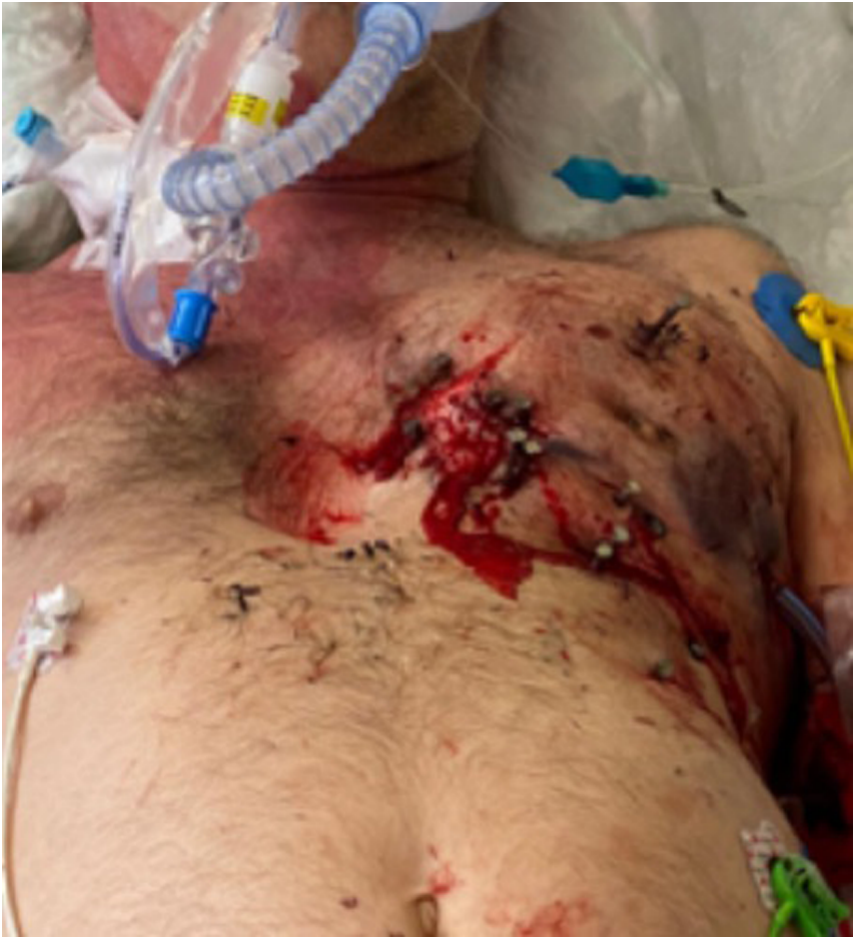
Multiple nail gunshots through the chest wall.

**Figure 2:**
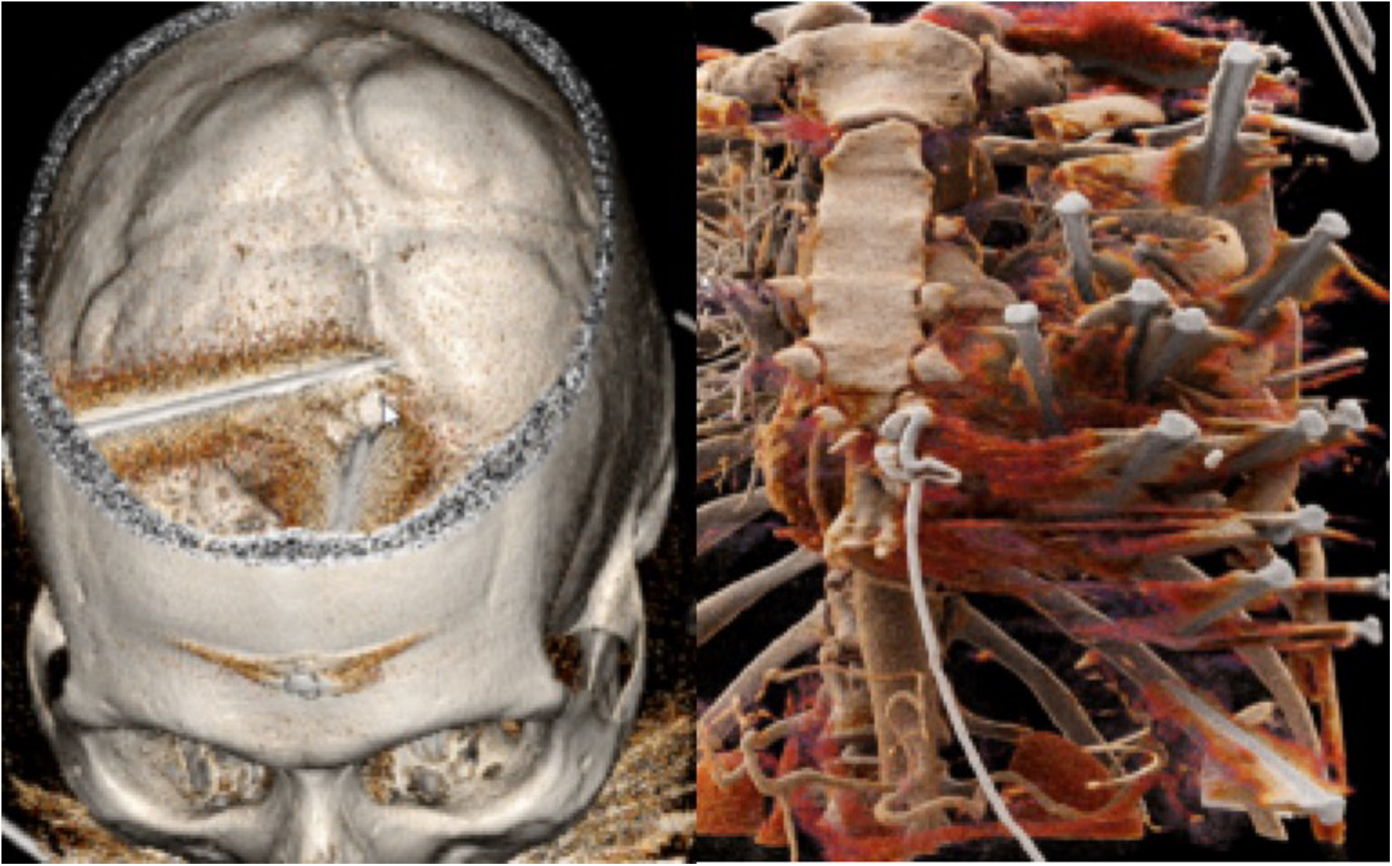
3d reconstruction of the CT-scan showing nails in the head (left) and those penetrating into the heart, the left lung and the stomach (right).

A 59-year-old male was admitted to the emergency department following a suicidal attempt using an automatic nail gun less than 1 h before. He presented with a Glasgow scale of 15 and was in a hemodynamically stable condition. Inspection revealed multiple nails penetrating the head, the neck, the thorax and the left upper abdomen (Figure 1). Total body computer tomography was performed (Figure 2) and demonstrated eighteen 7.5 cm long nails: two nails penetrated the skull, one from left paramedian and the other from right parietal causing a small subarachnoid and a thin subdural hematoma with a small amount of blood in both ventricles. One nail penetrated the neck at the level of the 7^Th^ cervical vertebra with the tip close to the left subclavian artery. Ten nails penetrated the left chest with the tips both in the right and left ventricle, some of them were shot through the interventricular septum. As expected, pericardial effusion was noticed but without signs of tamponade. Two nails penetrated the left chest: one superficially into the pectoralis muscle and the other one into the lingula lobe of the left lung. Finally, three nails entered the upper abdomen and perforated the stomach with a small amount of sub-diaphragmatic air. The patient was discussed at an interdisciplinary meeting including all concerned medical specialists to decide on the optimal sequence of surgical explorations.

The main concerns were the following:

• occurrence of cerebral bleeding following removal of the intracranial nails,
• pericardial tamponade if removal of the intracardiac nails would be delayed,
• need for full heparinization with the potential of delayed cerebral bleeding in case extracorporeal circulation would be required to allow safe cardiac repair.

Finally, decision was taken to remove the nails from the skull first and to proceed thereafter with chest and especially cardiac exploration since the patient was in a hemodynamic stable condition. The procedure was performed in a multipurpose trauma operating room, in which immediate cardiac exploration would have been possible.

**Figure 3:**
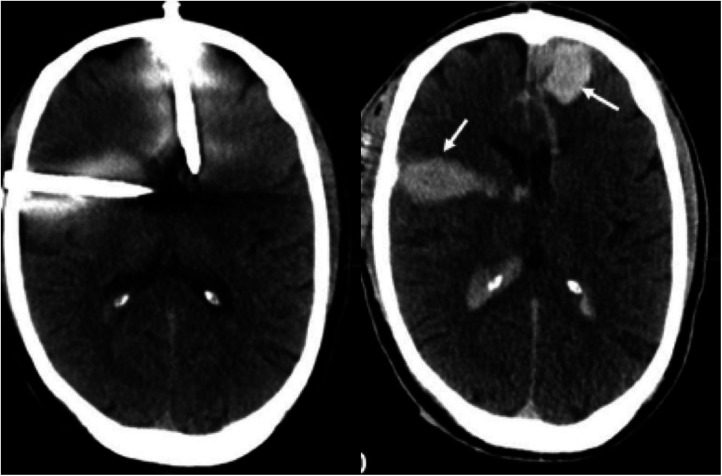
Cranial CT-scan before removal of the nails from the skull (left) with discrete bleeding and after removal with a progressive hematoma (arrow) (right).

Following removal of the nails from the head, decision was made to observe the situation for a short interval. Cerebral CT-scan was repeated and showed a slight increase of the hematoma in the left frontal and right temporal regions (Figure 3). Since echocardiography did not show any increase of pericardial effusion, the patient was transferred to the intensive care unit and reassessed 2 and 4 h later. There, during careful removal of the clothes that had been transfixed by the nails, the patient's condition suddenly deteriorated very rapidly and echocardiography showed pericardial tamponade. Because of circulatory collapse, mechanical resuscitation would have been necessary but chest compression was not possible because of the nails still *in situ*. For this reason, emergency left thoracotomy was performed on the intensive care unit but adequate manual cardiac massage was not possible with the nails still *in situ*.

**Figure 4:**
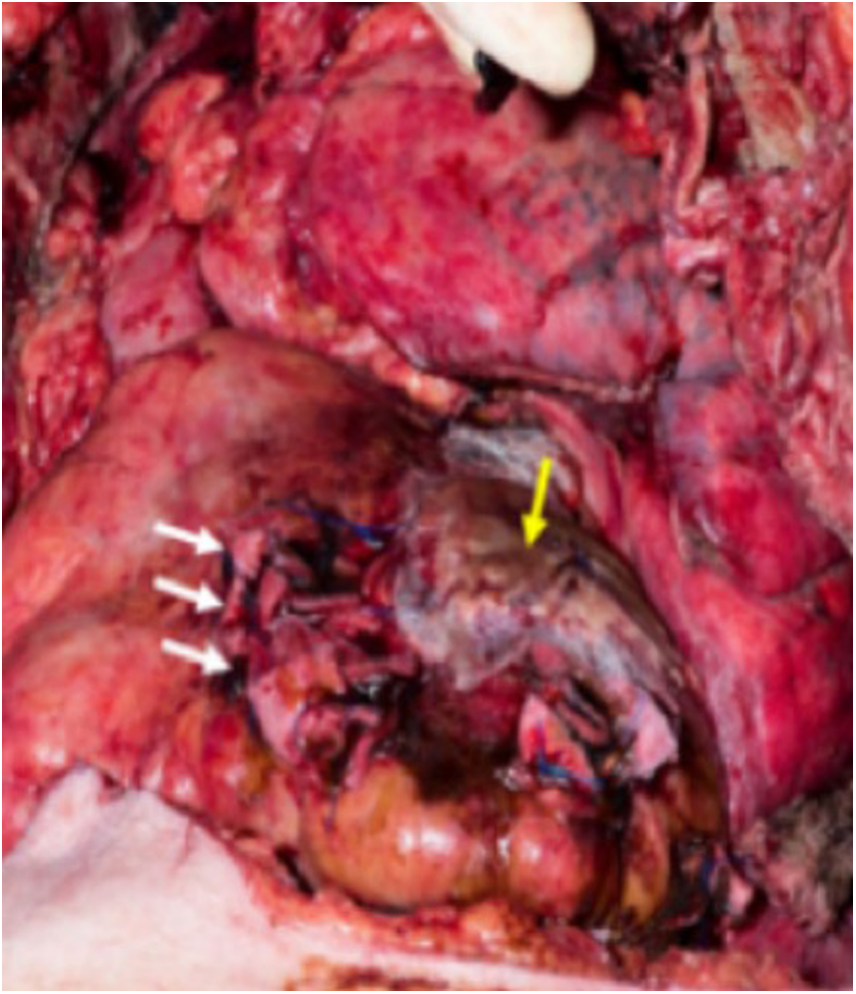
Intraoperative picture during repair of the right ventricular lesions (white arrows) and pericardial patch covering the left ventricular lesion (yellow arrow).

Opening the pericardium allowed immediate decompression of the tamponade. Following removal of the nails, quick inspection revealed seven myocardial lesions, some of them very close to the left anterior descending branch. Median sternotomy to improve exposure and allow better access to all injuries was performed: during that time, control of the most important myocardial injuries was performed with fingers and swabs. The lesions of the ventricular wall were closed using 2.0 polypropylene, while a diffuse epicardial venous bleeding was covered with a xeno-pericardial patch under which bio-glue (CryoLife Inc. GA, USA) was injected (Figure 4). Despite sufficient hemostasis within the chest, the patient remained unstable. Median laparotomy was performed to exclude intra-abdominal bleeding; this allowed removal of the last two nails that had perforated the stomach. These injuries were sutured with 4.0 Polydiaxone. The patient received 6 units of red blood cells.

Because of the contaminated operative field and semi-sterile operative conditions, the sternotomy wound was covered with a vacuum-dressing and delayed chest closure was performed five days later. Antibiotic regime consisted in cephalosporine until the chest was closed and vancocin for 48 h. The patient required prolonged ventilation and tracheotomy for weaning from ventilator. He was discharged on postoperative day 28, with full recovered motoric and cognitive functions.

**Figure 5:**
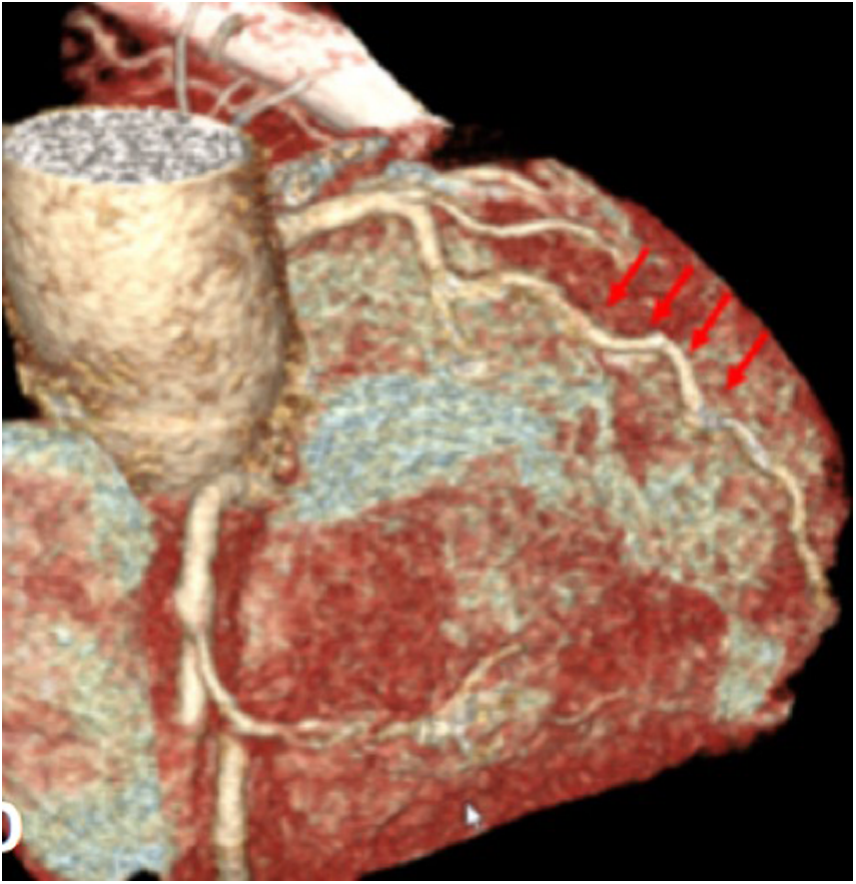
Postoperative CT scan showing the patent left anterior descending artery (red arrows).

The pre-discharge echocardiography showed normal biventricular function without valvular lesion or intracardiac shunts. Cardiac CT-scan showed patent coronary arteries (Figure 5).

## Comment

Combined penetrating cerebral and cardiac injuries constitute a particular challenge for decision-making regarding the optimal sequence of surgical explorations (1, 2). Especially patients who may need cardiopulmonary bypass and full heparinization have to be considered very carefully in presence of concomitant cerebral and/or abdominal injuries. In case of polytrauma and unstable hemodynamics, the lesion with the highest blood loss has to be treated first. Whenever possible, repair of cardiac injuries should be attempted without cardiopulmonary bypass to avoid full heparinization in a situation with multiple injuries. Adenosine administration to decrease pulse rate and blood pressure may be helpful (3). When this is not possible, heparinization should be quickly reversed by protamine in a 1 : 1 dosage to minimize the bleeding risk. Since simple algorithms for such situations are not available, multi-disciplinary evaluation of each individual case is of utmost importance to balance advantages and disadvantages of the different operative sequences.

In this case, the decision to proceed with cerebral extraction of the nails first and delay explorative thoracotomy was suboptimal since it led to an emergency situation on the intensive care unit and cardiac massage was not possible until the nails were removed. Nevertheless, the situation could be controlled and the final control of all cardiac injuries was realized through sternotomy.

In addition, postponing the removal of the intra-cranial nails would probably have avoided the progression of cerebral bleeding. Extraction could have been performed after optimization of the coagulation.

The choice of the optimal access to the heart was also an important aspect in this patient. Although median sternotomy is usually suitable for a single penetrating injury, it was thought inappropriate in this case because the nails *in situ* would have made spreading of the sternum impossible before removal of the nails.

Fortunately, the nails could be removed and the myocardial lesions successfully treated without the need for extracorporeal circulation. If this would not have been possible either ECMO or CPB with coated tubes would have been attractive options to avoid full heparinization. Despite all challenges encountered, this case ended successfully without wound infection despite the hardly sterile operative conditions and the patient could be discharge at home with a psychiatric supervision.

## Data Availability

The raw data supporting the conclusions of this article will be made available by the authors, without undue reservation.
